# Research on the improvement of CPI basic classification index compilation method in digital economy

**DOI:** 10.1371/journal.pone.0322465

**Published:** 2025-05-07

**Authors:** Zhixiu Du, Jinzhu Du

**Affiliations:** 1 School of Statistics and Mathematics, Inner Mongolia University of Finance and Economics, Inner Mongolia, China; 2 Inner Mongolia University of Finance and Economics, Inner Mongolia, China; Guilin University of Aerospace Technology, CHINA

## Abstract

Information and communication technology (ICT) products are the core of the digital economy, and their classified price index plays an important role in the compilation of CPI index. This paper starts from the characteristics of ICT products that have a fast update rate and do not necessarily meet the unit substitution elasticity between products, and improves the traditional product price index model by considering the mismatch item processing and product substitution elasticity and chain drift factors to construct the Hedonic-SV-RYGEKS price index model in this paper. Using the weekly data of Jingdong mobile phone price on whale staff platform and the monthly data of notebook computer on magic mirror insight platform, after processing, a total of 1586 sets of mobile phone data and 136 sets of notebook computer data are obtained. By writing SPSS macro program and python program, the weekly price index of mobile phone and the monthly price index of notebook computer are calculated, and the ring price index and fixed base price index of mobile phone and notebook computer are compiled respectively. The chain ring price index based on model calculation is compared with the fixed base price index to investigate the rationality of the model. The results show that: Firstly, based on the principle of the quality adjustment model, the characteristic variables that can reflect the characteristics of the product are selected, and a Hedonic quality adjustment model is established between them and the product price. Through the actual data test, the model is suitable for fitting the price of mismatched products. Secondly, from the perspective of reflecting the elasticity of substitution of products, the evaluation criteria of the price index, and the adjustment of product quality, this paper constructs the Hedonic-SV-RYGEKS price index based on the Hedonic model and SV index, which avoids the incomparability of samples caused by the low matching degree of inter-temporal samples, and effectively inhibits the chain drift of chain price index caused by the rapid update of products. Finally, it is hoped that the research content of this paper can provide a reference for improving and innovating the processing method of mismatched projects in the compilation of price index.

## Introduction

ICT technology, as the core of the digital economy, is becoming an important engine to promote the development of new productivity boosters. ICT products are different from other products, and their quality will change greatly in a short period, which determines that the traditional price index method can not be used to measure the price change. Therefore, it is of great practical significance to compile the price index [1] of ICT products that can more effectively reflect the market supply and demand.

### Foreign research on price index quality adjustment and GEKS index

The two problems in estimating price indices brought about by the digital economy are manifested as the impact of “chain drift” and “product substitution elasticity” on the calculated index values. This issue can be confirmed from international CPI manuals and scholar research. In terms of international standards, as early as the international standard ILO [2], it was pointed out that as the time period becomes shorter, chained indices exhibit more “drift”. There is cross substitution elasticity between products, although it is likely to be difficult to obtain a satisfactory, acceptable estimate of the numerical value of the elasticity of substitution. The ILO [3] after 16 years indicates that high frequency chaining of weighted price indices, including superlative price indices, can lead to strong chain drift. And a dedicated chapter was set up to delve into the issue of project substitution, particularly the methods of incorporating new products into the index. Eurostat [4] elaborates the multilateral method of estimating consumer price index. Erwin [5] mentioned at the 30th Anniversary of the Ottawa Group conference that chain drift problem in scanned data, consumer price index theory include stochastic approaches, quality adjustment methods, and a Consumer Price Index should probably take into account substitution effects and so on.

In terms of scholar research, regarding the suitability of fixed basket representative specifications, traditional price index estimation suffers from chain drift issues, Ivancic et al. [6], de Haan et al. [7] empirical research on scanner data shows that under the action of high-frequency chains, the optimal index shows obvious chain drift. In order to solve the problem of chain drift, Ivancic et al. [6] used the GEKS method proposed by Gini [8], Eltetö et al. [9] and Szulc [10] to apply the price comparison between countries to the price comparison across time. In terms of changes in the quality of new and old products, de Haan et al. [11] proposed an interpolation method of Imputation Trnqvist Rolling Year GEKS (ITRYGEKS) index, which is suitable for the case of complete product characteristics information. However, supermarket scanner data and online data usually do not contain enough characteristic information, which limits the use of this method. Compared with ITRYGEKS, the fixed effect index can be used to deal with the quality adjustment problem of incomplete characteristics information of products. Krsinich [12] used the fixed effect window splicing method to study this problem. Białek [13] pointed out that select the price index formula could reduce a chain drift. Knížat et al. [14] developed bilateral and multilateral price indices for refrigerator product categories and compared the two. Peter et al. [15] proposed a new splicing extension multilateral indices method against the decomposability of GEKS index, and compiled price index using web scraped data, which obtained from the Slovak market.

The question of whether the substitution elasticity between specifications is 1. Different from the traditional sparse sampling sample data, the digital economy era may require well-representative spot-shelf price data. Whether the elasticity of substitution between products is 1 remains to be tested, Diewert [16] introduced the constant elasticity of substitution (CES) utility function as the basis for the construction of the target cost of living index. Ian et al. [17] developed a new method of price-quality adjustment by using the CES index and quality adjustment method and applied it to the compilation of the British automobile sales price index. However, there are not many articles on the estimation of the elasticity of substitution of scanned products. Can [18] using the scanning data of soda, dairy products, coffee and cheese estimating the product elasticity of substitution. Diewert et al. [19] simulated and calculated the substitution deviation of different multilateral methods, suggested using the Caves–Christensen–Diewert–Inklaar index with a new method. Jeon et al. [20] showed that scanner data generate different elasticities than other data types. Jacek etal. [21] calculated the CES substitution elasticity of the product using scanned data and compiled the CES cost of living index, also verifies how the elasticity of substitution estimates affect the differences between the values of the CES indices.

### China research on price index quality adjustment and GEKS index

In the domestic research on the multilateral price index in the era of the digital economy, the representative literature is: Chen Lishuang et al. [22] conducted an extensive study on the chain drift and window width selection of the GEKS index. Chen Menggen et al. [23] made full use of the advantages of big data to adjust and improve CPI statistics in data collection, calculation methods, weight selection, seasonal adjustment, quality adjustment, data publication, and other aspects. Chen Lishuang et al. [24] reviewed the construction method of GEKS, discussed the updating method of GEKS index sequence and the selection of moving window width in CPI compilation, and discussed the cross-substitution elasticity between products in the digital economy era. The constant elasticity price index was derived and compared with the GEKS index. Lei Zekun et al. [25] proposed a weighted nonlinear hedonic price model that fully considers the scale effect of characteristic variables and the economic significance of the model, and solved the chain drift with the help of the rolling year GEKS index and carried out a trial calculation using the big data of Jingdong platform. Chen Lishuang et al. [26] proposed a method to construct a drift-resistant flexible commodity basket by programming, which can solve the drift of high-frequency big data chain price index. Xu Xianchun et al. [27] comprehensively explained the main updates of the 2020 edition of CPI compilation manual, and put forward the enlightenment of the manual update to China ‘s CPI compilation. Xu Qiang et al. [28] analyzed the challenges of traditional CPI compilation methods brought by the rapid replacement of products and the endless emergence of new business models in the digital economy era and gave suggestions for CPI compilation.

Looking at the research of scholars at home and abroad, there are many literatures on index compilation mainly from the aspects of ‘dynamic base period’ of price index compilation and price index chain drift. There are not many literatures on the price index considering the high loss rate of products and the elasticity of product substitution. The purpose of this paper is to construct a Hedonic-SV-RYGEKS price index model considering the incomparability of inter-temporal samples, the elasticity of substitution between products and the chain drift of price index by referring to the suggestion of ‘Consumer Price Index Manual: Concepts and Methods (2020)’. Based on the price data of mobile phones and notebook computers with different frequencies obtained by free ‘crawler’, the corresponding price indexes of mobile phones and notebook computers are compiled. It further confirms the operability and feasibility of the Hedonic-SV-RYGEKS price index model. It provides a reference for solving the sample incomparability caused by the high loss rate of products in the compilation of CPI basic classification index in the era of digital economy and improving the accuracy of price index compilation.

Specifically, the main possible contributions of this paper are:

Firstly, referring to the suggestion of ‘Consumer Price Index Manual: Concepts and Methods (2020)’, the Hedonic-SV-RYGEKS price index model is constructed, and how the model adjusts the quality of products is discussed. How to reduce the mismatch of inter-temporal samples, how to reflect the elasticity of substitution between products and how to reduce the chain drift of chain price index are discussed. The accuracy and feasibility of compiling price index by this model are demonstrated theoretically.

Secondly, based on the constructed Hedonic-SV-RYGEKS model, the online price data of mobile phone’s ‘weekly price’ and notebook computer’s ‘monthly price’ are obtained through ‘crawler’, and the linear Hedonic-Robust/ Huber-SV-RYGEKS price index model of mobile phone and the semi-logarithmic Hedonic-WLS-SV-RYGEKS price index model of notebook computer are established to compile the Hedonic-SV-RYGEKS price index of mobile phone and notebook computer. The difference between the two models lies in the different selection forms of the Hedonic model and the different treatment methods for the data heteroscedasticity phenomenon.

## Hedonic-SV-RYGEKS price index construction method for mismatched items

### Hedonic price estimation model

The Hedonic adjustment method is a widely used quality adjustment method. The basic idea of this method is that the consumer demand for the product is not based on the product itself, but on the physical characteristics of the product, and the price of the product is determined by these physical characteristics. The Hedonic model for price index quality adjustment model includes three forms: linear model, semi-logarithmic model and logarithmic model. The linear model representation is shown in Eq (1).


$$p_{i}=\alpha +\sum_{j}\beta_{j}z_{ij}+\varepsilon_{i}$$ (1)

where, $p_{i}$ is the price of the *i*-th product, α is the constant term, $\beta_{j}$ is the characteristic marginal price, and $z_{ij}$ is the *j*-th characteristic of the *i*-th product.

### Constant Substitution Elasticity (CES) index

The CES index was first proposed by Lloyd [29] and Moulton [30], Stephen et al. [31] gave a CES index in which consumer preferences change over time. Another arithmetic mean expression of the CES index is the Eq (2). The Eq (2) is a CES function form of consumer preference changing with time.


$$P_{jk}^{CES}\text{=}{\left[\sum_{i}{\left(\frac{\eta_{ik}}{\eta_{ij}}\right)}^{\sigma -1}\cdot s_{j}^{i}\cdot {\left(\frac{p_{ik}}{p_{ij}}\right)}^{1-\sigma }\right]}^{\frac{1}{1-\sigma }}$$ (2)

where, the subscripts ‘*j*’ and ‘*k*’ represent the base period and the reporting period respectively, $P_{jk}^{CES}$ is the CES index, $P_{i}$ is the price of the *i*-th product, σ is the elasticity of substitution caused by relative price changes between products, *i* represents the product category (*i* = 1, 2,..., m), $s^{i}$ is the share of expenditure of the *i*-th product, and $\eta_{i}$ is the consumer preference parameter of the *i*-th product.

### Sato-Vartio index

The CES index shown in the Eq (2) has incomparable advantages over other indexes in reflecting product elasticity of substitution and consumer preference, but it is not transferable and cannot be used in multilateral comparisons.

The Sato-Vartia (SV) index is an index proposed by Sato-Vartia [32] to measure price and volume changes in the form of a logarithmic index. The specific expression is shown in Eq (3) to Eq (5). Compared with the CES index, the elasticity of substitution in the index is calculated when the consumer preference does not change with time, so the SV index can be considered as one of the CES index. According to Stephen et al. [31], the relationship between SV index and CES index can be expressed by Eq (6).


$$P_{jk}^{SV}=\prod_{i=1}^{N}{\left(\frac{p_{ik}}{p_{ij}}\right)}^{\varphi_{i}^{jk}}$$ (3)

$$\varphi_{i}^{jk}=\frac{\left(\omega_{ik}-\omega_{ij}\right)/\left(\ln\left(\omega_{ik}\right)-\ln\left(\omega_{ij}\right)\right)\;}{\sum_{i=1}^{N}\left(\omega_{ik}-\omega_{ij}\right)/\left(\ln\left(\omega_{ik}\right)-\ln\left(\omega_{ij}\right)\right)\;}$$ (4)

$$\omega_{ik}=\frac{p_{ik}\cdot q_{ik}}{\sum_{i=1}^{N}p_{ik}\cdot q_{ik}}$$ (5)

$$\ln\left(p_{jk}^{CES}\right)=\ln\left(p_{jk}^{SV}\right)-\left[\sum_{i}\varphi_{ik}\cdot \ln\left(\frac{\eta_{ik}}{\eta_{ij}}\right)\right]$$ (6)

where, ln(·) is the natural logarithmic function, $p_{jk}^{SV}$ is the SV index, $\omega_{ik}$ is the expenditure share of the *k*-th period of the *i*-th product, $\eta_{ik}$ and $\eta_{ij}$ are the consumer preference parameters of the *k*-th and *j*-th period of the *i*-th product, respectively. The Eq (6) shows that the CES index is equal to the SV index minus the consumer preference bias. It can be seen that when the consumer preference parameter vector $\ln\left(\eta_{ik}/\eta_{ij}\;\right)$ is orthogonal to $\varphi_{i}^{jk}$, the SV index is equal to the CES index.

In summary, the SV index can not only reflect the elasticity of substitution between products, but also satisfy the nature of the superior index. The SV index satisfies the transitivity when the weight parameter $\varphi_{i}^{jk}$ of each option is the same. Therefore, this paper considers the SV index that satisfies the transitivity as the basic price index for constructing the GEKS index. The specific equations are shown in Eqs (7) and (8).


$$P_{jk}^{SV}=\prod_{i=1}^{N}{\left(\frac{p_{ik}}{p_{ij}}\right)}^{\varphi_{i}}$$ (7)

$$\varphi_{i}^{jk}=\varphi_{i}=\sum_{i=1}^{n}\sum_{j=1}^{n}\varphi_{i}^{jk}/C_{n}^{2}\;$$ (8)

### Rolling Year GEKS (RYGEKS) model

Ivancic et al. proposed the rolling year GEKS price index. This method solves the problem of continuous correction of the index, and also solves the problem of deviation of the optimal index chain. The specific calculation is shown in Eq (9).


$$P_{0,T+1}^{RYGEKS}=P_{0,T}^{GEKS}\cdot P_{T,T+1}^{RYGEKS}$$ (9)

where, $P_{0,T}^{GEKS}$ and $P_{0,T+1}^{RYGEKS}$ represent the GEKS price index of period 0 to period *T* and period 0 to period *T* + 1, respectively, and $P_{T,T+1}^{RYGEKS}$ represents the GEKS price index of period *T* to period *T* + 1.

After the above analysis, this paper constructs the GEKS index with the SV index as the basic price index for the price after quality adjustment, as shown in Eq (10).


$$P_{jk}^{SV-GEKS}=\prod_{l=1}^{N}{\left[P_{jl}^{SV}\cdot P_{lk}^{SV}\right]}^{1/N}$$ (10)

From Eqs (9) and (10), the Eq (11) for calculating the rolling annual fixed base price index is obtained:


$$P_{0,T+1}^{SV-RYGEKS}=P_{0,T}^{SV-GEKS}\cdot P_{T,T+1}^{SV-RYGEKS}$$ (11)

In summary, this paper calls this method of compiling the classified price index of CPI information and communications products as the ‘Hedonic-SV-RYGEKS’ method. This method not only considers the high turnover rate of ICT products, but also considers the impact of substitution elasticity between products on prices, and also considers the chain drift problem of chain price index compilation. In a word, in theory, this index compilation model is a good model, but it still needs further empirical test to prove its practical feasibility.

## An empirical study on the compilation of mobile phone price index based on Hedonic-SV-RYGEKS index

### Data sources and data processing

This section chooses the basic classification of mobile phones in ICT products as the research object and compiles its corresponding price index. Due to the lack of commodity trading volume information, the data resources obtained by the ‘crawler’ are not suitable for the research of this paper. The sales data of specific products related to e-commerce are generally not open to the public, so it is difficult to obtain specific sales data related to e-commerce. Considering the availability of data, this paper obtains 57 consecutive weeks, 5700 price and sales volume, and sales data of all mobile phone brands in the top 100 from August 2022 to September 2023 through Jingdong mobile phone transaction data resources. Because there is no specific information about the quality of the mobile phone in the mobile phone transaction data resources of the whale staff, there is only a URL link to the details of the mobile phone. According to the details of the mobile phone on the web page, the information selected in this paper to reflect the quality of the mobile phone also includes CPU model, running memory, fuselage memory, rear main pixel, and screen size. These data are obtained by Python programming through the corresponding URL links provided above.

In view of the weekly data used in this paper, in terms of window width selection, referring to Frances [12], the window width is selected to be 53 weeks, that is, the first 53 weeks of the 57 weeks are used as window widths, and the remaining 4 weeks are used for rolling calculation of the fixed base price index. Firstly, the 53 weeks data is grouped, and any two weeks are used as a group to examine the mobile phone matching. Since the data here is the top 100 mobile phone-related data per week, from the comparison of mobile phones every two weeks, mobile phone products are ‘updated’ quickly, ‘old products’ disappear more, and ‘new products’ appear more, resulting in more mismatched items within two weeks of comparison. In order to solve the problem of price comparability of mobile phones in any two weeks, it is considered that the price difference of mobile phones in any two weeks does not match the quality factor. For example, the first week ‘s mobile phone is Apple ‘s mobile phone, and the second week ‘s mobile phone is Huawei ‘s mobile phone. The price difference between the two mobile phones contains quality factors, not purely price factors. Therefore, this paper argues that if the quality factor can be eliminated, then Apple ‘s mobile phone and Huawei ‘s mobile phone are comparable in these two weeks, which is equivalent to the ‘fixed goods’ in the ‘fixed basket goods’ in the price index compilation. Based on this idea, the quality adjustment model is used to adjust the price of mismatched mobile phones in any two weeks to obtain a comparable mobile phone price sequence in any two weeks.

Through the above process, the new sample data calculated by the price index in this paper are obtained. The sample size of mobile phones per week is still 100, and any two weeks of mobile phones are comparable after quality adjustment.

### Compilation of mobile phone price index based on Hedonic-SV-RYGEKS method

#### Processing of mismatched items based on linear Hedonic model.

According to Eq (1), the regression modeling of the average price of 57 weeks mobile phones and its related characteristic variables (characteristic variables include running memory, fuselage memory, screen size, after-action main pixel and CPU model) is carried out. The CPU model is treated as a qualitative variable and divided into 45 types of CPU, which is introduced into the model as a dummy variable. The model does not consider the constant term and uses the ‘Backward’ method for regression. For the weekly data with heteroscedasticity, Robust regression or Huber regression is used to correct the OLS regression”. The estimation of 57 groups of equations is realized by writing SPSS macro program. The goodness of fit and significance test of Hedonic regression equation are shown in Tables 1 and 2. The corresponding regression coefficients of the 57 sets of equations established by passed the test at the significance level α = 0.1. Due to the long length of the regression coefficients, only part of the week’s regression coefficients are intercepted here, as shown in Table 3.

**Table 1:** From August 2022 to September 2023, the average price of mobile phones for 57 weeks and its characteristic variable regression equation goodness of fit.

Weeks	Model	R^2^	Adjusted R^2^	Weeks	Model	R^2^	Adjusted R^2^	Weeks	Model	R^2^	Adjusted R^2^	Weeks	Model	R^2^	Adjusted R^2^
202231	15	0.973	0.970	202246	12	0.831	0.810	202308	23	0.755	0.745	202322	19	0.746	0.729
202232	18	0.916	0.911	202247	18	0.831	0.817	202309	21	0.687	0.674	202323	16	0.699	0.660
202233	16	0.982	0.981	202248	21	0.775	0.760	202310	19	0.762	0.749	202324	10	0.702	0.642
202234	16	0.984	0.982	202249	16	0.769	0.747	202311	16	0.825	0.807	202325	18	0.733	0.713
202235	12	0.983	0.981	202250	24	0.786	0.780	202312	10	0.836	0.800	202326	22	0.688	0.668
202236	14	0.984	0.982	202251	5	0.826	0.783	202313	16	0.773	0.750	202327	26	0.634	0.623
202237	6	0.991	0.989	202252	20	0.824	0.808	202314	19	0.804	0.793	202328	23	0.628	0.613
202238	13	0.983	0.981	202301	19	0.789	0.773	202315	11	0.839	0.815	202329	26	0.611	0.599
202239	13	0.991	0.990	202302	20	0.831	0.820	202316	26	0.687	0.674	202330	17	0.704	0.675
202240	11	0.991	0.990	202303	22	0.774	0.765	202317	20	0.724	0.703	202331	22	0.634	0.610
202241	12	0.992	0.991	202304	19	0.782	0.768	202318	21	0.735	0.712	202332	22	0.627	0.612
202242	10	0.996	0.995	202305	21	0.799	0.786	202319	23	0.655	0.636	202333	23	0.672	0.648
202243	10	0.996	0.995	202306	18	0.834	0.821	202320	26	0.636	0.625	202334	24	0.637	0.613
202244	12	0.993	0.992	202307	23	0.781	0.774	202321	20	0.720	0.696	202335	23	0.666	0.644
202245	14	0.987	0.986												

**Table 2:** The significance of the regression equation between the average price of mobile phones and its characteristic variables in 57 weeks from August 2022 to September 2023.

Weeks	Models	F	Significance	Weeks	Models	F	Significance
202231	15	366.37	0.00	202246	12	39.77	0.00
202232	18	171.16	0.00	202247	18	56.71	0.00
202233	16	644.11	0.00	202248	21	53.84	0.00
202234	16	537.53	0.00	202249	16	33.73	0.00
202235	12	476.29	0.00	202250	24	119.01	0.00
202236	14	556.01	0.00	202251	5	19.01	0.00
202237	6	476.80	0.00	202252	20	53.69	0.00
202238	13	465.69	0.00	202301	19	49.72	0.00
202239	13	891.00	0.00	202302	20	76.96	0.00
202240	11	884.91	0.00	202303	22	82.22	0.00
202241	12	825.31	0.00	202304	19	56.06	0.00
202242	10	1473.56	0.00	202305	21	62.14	0.00
202243	10	1473.09	0.00	202306	18	66.62	0.00
202244	12	1205.96	0.00	202307	23	114.07	0.00
202245	14	847.83	0.00	202308	23	74.08	0.00
							
202309	21	52.80	0.00	202324	10	11.53	0.00
202310	19	60.71	0.00	202325	18	36.43	0.00
202311	16	47.52	0.00	202326	22	34.54	0.00
202312	10	23.22	0.00	202327	26	55.97	0.00
202313	16	34.35	0.00	202328	23	40.59	0.00
202314	19	76.20	0.00	202329	26	50.78	0.00
202315	11	34.50	0.00	202330	17	24.04	0.00
202316	26	52.78	0.00	202331	22	27.10	0.00
202317	20	34.49	0.00	202332	22	40.39	0.00
202318	21	31.87	0.00	202333	23	27.26	0.00
202319	23	35.63	0.00	202334	24	27.44	0.00
202320	26	56.45	0.00	202335	23	30.27	0.00
202321	20	29.60	0.00				
202322	19	45.48	0.00				
202323	16	18.14	0.00				

**Table 3:** Coefficients of the regression equation (Robust/ Huber) of the average price of mobile phones and its characteristic variables in the 31 st and 32 nd weeks of 2022.

Weeks	Model	B	Standard error	z	Significance	Interval-OLS	Interval-Robust/HuberT
202231	RAM	145.957	35.422	4.120	0.000	[83.432, 208.481]	[75.595, 216.318]
	ROM	6.127	1.895	3.234	0.002	[3.316, 8.938]	[2.363, 9.891]
	Aftertaking main pixel	-0.082	0.022	-3.769	0.000	[-0.133, -0.032]	[-0.126, -0.039]
	Tiangi 700 series	-667.832	160.558	-4.159	0.000	[-1329.877, -5.787]	[-986.760, -348.904]
	Tiangi 800 series	-418.277	127.505	-3.280	0.001	[-907.015, 70.462]	[-671.550, -165.003]
	Tiangi 900 series	-603.806	156.925	-3.848	0.000	[-1273.039, -37.074]	[-915.518, -292.094]
	Snapdragon 870	-653.191	239.657	-2.726	0.008	[-1269.307, -37.074]	[-1129.240, -177.141]
	A15	5782.352	328.822	17.585	0.000	[5377.504, 6187.200]	[5129.187, 6435.518]
	Apple A series	3782.377	191.213	19.781	0.000	[3152.186, 4412.568]	[3402.555, 4162.199]
202232	ROM	12.472	1.049	11.887	0.000	[10.389, 14.556]	[10.416, 14.529]
	Aftertaking main pixel	-0.091	0.037	-2.458	0.014	[-0.164, -0.017]	[-0.163, -0.018]
	Snapdragon 870	-848.615	417.402	-2.033	0.042	[-1677.376, -19.835]	[-1666.707, -30.522]
	UNISOC	1642.955	660.040	2.489	0.013	[332.429, 2953.480]	[349.299, 2936.610]
	A15	4530.306	310.970	14.568	0.000	[3912.867, 5147.744]	[3920.816, 5139.796]
	Apple A series	3627.937	494.717	7.333	0.000	[2645.664, 4610.209]	[2658.309, 4597.564]

It can be seen from Tables 1 and 2 that the goodness of fit of the regression model for these 57 weeks is above 0.6. Therefore, the overall model fitting effect is good, and the equations all pass the significance test.

The data of any two weeks in the first 53 weeks are matched according to the characteristic variables. The characteristic variables corresponding to the unmatched items in the week of any two weeks are brought into the Hedonic regression model corresponding to the week of 2, and the estimated price of this kind of products in the week of 2 is calculated. A total of 1378 sets of estimated prices need to be calculated. This process is realized by writing SPSS macro program. Taking the data of the 31st and 32nd weeks of 2022 as an example, the processing results of fully matched and mismatched items are shown in Tables 4 and 5.

**Table 4:** 31st week and 32nd week of 2022 exactly match the price unit of the mobile phone: Yuan.

Weeks	Mobile phone brand	Average price of the first week	Average price in second week	Weeks	Mobile phone brand	Average price of the first week	Average price in second week
202231	HUAWEI	1599	1699	202231	K-Touch	129	129
202231	NOKIA	149	149	202231	K-Touch	99	99
202231	NOKIA	249	299	202231	MI	649	699
202231	Apple	4099	4099	202231	MI	699	699
202231	Apple	4899	4899	202231	MI	2149	2149
202231	Apple	4899	4899	202231	MI	2799	2799
202231	Apple	5999	5999	202231	MI	1199	1199
202231	Apple	5999	5999	202231	MI	1399	1399
202231	Apple	6799	6799	202231	MI	1899	1899
202231	Apple	8799	8799	202231	MI	999	999
202231	Apple	8999	8999	202231	OPPO	2499	2499
202231	Apple	9799	9799	202231	OPPO	1399	1399
202231	HONOR	899	899	202231	vivo	2099	2099
202231	HONOR	1199	1149	202231	vivo	2499	2499
202231	HONOR	1199	1149	202231	vivo	1499	1499
202231	HONOR	1569	1699				

Note: The first week indicates 31nd week of 2022, or 202231, and the second week indicates 32nd week of 2022, or 202232. The following table is the same.

**Table 5:** The average price and fitting price unit of the mismatched items in 31st week and 32nd week: yuan.

Weeks	mobile phone brand	Average price of the first week	Fit Price for second week	Weeks	mobile phone brand	Average price of the first week	Fit Price for second week	Weeks	mobile phone brand	Average price of the first week	Fit Price for second week
202231	PHILIPS	228	2069	202231	Apple	7999	6250	202231	MI	2149	2416
202231	HUAWEI	999	5713	202231	Apple	8799	7640	202231	MI	2799	3849
202231	HUAWEI	999	5713	202231	Apple	8799	7640	202231	MI	2799	3849
202231	HUAWEI	2999	2384	202231	Apple	8999	6250	202231	MI	2299	836
202231	HUAWEI	1599	2042	202231	Apple	9799	7640	202231	MI	2499	1909
202231	HUAWEI	1599	2042	202231	HONOR	2199	2384	202231	MI	3299	8176
202231	HUAWEI	4588	4408	202231	HONOR	2198	2384	202231	MI	1039	1892
202231	Apple	4099	4632	202231	HONOR	2698	2499	202231	MI	1199	850
202231	Apple	4899	4632	202231	HONOR	1199	1387	202231	MI	1199	850
202231	Apple	4899	4632	202231	HONOR	1199	1641	202231	MI	1369	1387
202231	Apple	5999	5713	202231	HONOR	1399	3032	202231	MI	1569	2777
202231	Apple	5999	5713	202231	HONOR	1049	3837	202231	MI	999	1120
202231	Apple	5999	5713	202231	HONOR	1049	3837	202231	MI	829	48
202231	Apple	6799	7103	202231	HONOR	1369	1039	202231	Ace	2999	5481
202231	Apple	6799	7103	202231	HONOR	1569	1575	202231	OPPO	1599	994
202231	Apple	6799	7103	202231	HONOR	1569	1575	202231	OPPO	1799	2384
202231	Apple	6799	7103	202231	K-Touch	159	155	202231	OPPO	1399	1387
202231	Apple	6499	7103	202231	K-Touch	99	155	202231	OPPO	1599	2777
202231	Apple	6799	7103	202231	K-Touch	99	155	202231	vivo	3999	3299
202231	Apple	5199	5713	202231	MI	649	660	202231	vivo	1799	1343
202231	Apple	5199	5713	202231	MI	649	660	202231	vivo	2099	1343
202231	Apple	7999	6250	202231	MI	699	1355	202231	vivo	1499	1641
202231	Apple	7999	6250	202231	MI	2149	2416	202231	vivo	1099	1039

It can be seen from Table 4 that in the 31st and 32nd weeks of 2022, based on the above characteristic variables, the number of mobile phones that can be fully matched is 31. From Table 5, it can be seen that the number of mobile phones that do not match in these two weeks is 69, among which, the ‘second week fitting price’ column is the fitting price of the mobile phone that does not match in the 31st week of 2022 in the 32nd week.

In summary, after the adjustment of the Hedonic-Robust/ Huber regression model, we obtained a comparable mobile phone price sequence excluding quality factors, which can be used for the calculation of mobile phone substitution elasticity later.

#### Calculation of substitution elasticity of mobile phone after quality adjustment in two adjacent weeks.

In order to investigate whether the elasticity of substitution of mobile phones is a unit elasticity of substitution, the model is introduced with reference to the practice of Ivancic et al. (2010), as shown in Eq (12).


$$\begin{array}{c} \ln\frac{s_{m}}{{\bar{s}}_{1}}=\left(1-\sigma \right)\ln\left[\frac{p_{m}}{{\bar{p}}_{1}}\right]+\varepsilon_{m} \end{array}$$ (12)

where, σ is the elasticity of substitution of mobile phones, $s_{m}$ and $p_{m}$ are the expenditure share and price of the *m*-th mobile phone respectively, ${\bar{s}}_{1}$ and ${\bar{p}}_{1}$ are the geometric mean of the expenditure share and price of all kinds of mobile phones respectively, and $\varepsilon_{m}$ is the random disturbance term.

According to Eq (12), combined with the price data adjusted by the hedonic model, the parameters of each model are estimated by the least square method. After the model test, the estimated value of the elasticity of substitution $\sigma_{i}\left(i=1,2,\cdots ,56\right)$ of mobile phones is obtained, as shown in Table 6.

**Table 6:** Estimated value of mobile phone substitution elasticity.

Elasticity of substitution	Numerical value	Elasticity of substitution	Numerical value	Elasticity of substitution	Numerical value	Elasticity of substitution	Numerical value
$\sigma_{1}$	1.437	$\sigma_{15}$	1.604	$\sigma_{29}$	1.918	$\sigma_{43}$	1.487
$\sigma_{2}$	1.040	$\sigma_{16}$	1.657	$\sigma_{30}$	2.422	$\sigma_{44}$	1.814
$\sigma_{3}$	1.439	$\sigma_{17}$	1.867	$\sigma_{31}$	1.485	$\sigma_{45}$	1.547
$\sigma_{4}$	1.492	$\sigma_{18}$	1.155	$\sigma_{32}$	1.848	$\sigma_{46}$	1.644
$\sigma_{5}$	1.298	$\sigma_{19}$	3.735	$\sigma_{33}$	1.576	$\sigma_{47}$	1.725
$\sigma_{6}$	1.163	$\sigma_{20}$	2.115	$\sigma_{34}$	1.396	$\sigma_{48}$	1.609
$\sigma_{7}$	1.281	$\sigma_{21}$	1.170	$\sigma_{35}$	1.691	$\sigma_{49}$	1.880
$\sigma_{8}$	1.325	$\sigma_{22}$	3.056	$\sigma_{36}$	1.236	$\sigma_{50}$	1.895
$\sigma_{9}$	1.689	$\sigma_{23}$	1.918	$\sigma_{37}$	1.375	$\sigma_{51}$	2.026
$\sigma_{10}$	1.507	$\sigma_{24}$	2.172	$\sigma_{38}$	1.392	$\sigma_{52}$	1.637
$\sigma_{11}$	1.905	$\sigma_{25}$	1.358	$\sigma_{39}$	1.105	$\sigma_{53}$	1.830
$\sigma_{12}$	2.352	$\sigma_{26}$	1.714	$\sigma_{40}$	1.740	$\sigma_{54}$	1.559
$\sigma_{13}$	3.267	$\sigma_{27}$	1.959	$\sigma_{41}$	1.379	$\sigma_{55}$	2.072
$\sigma_{14}$	1.882	$\sigma_{28}$	2.081	$\sigma_{42}$	1.628	$\sigma_{56}$	1.522

From Table 6, it can be seen that the substitution elasticity of mobile phones in the adjacent two weeks is greater than 1, and the substitution elasticity values of each group show a certain fluctuation. This shows that in the process of compiling the mobile phone price index, the traditional unit substitution elasticity price model has some shortcomings. Based on this, we consider using a price index calculation model that can reflect the elasticity of substitution of products to compile a mobile phone price index.

#### Based on Sato-Vartio mobile phone price index calculation.

After the above quality adjustment, 1378 sets of fully matched mobile phone products are obtained. Based on these data, SV price index is considered when the substitution elasticity of mobile phone does not meet the unit substitution elasticity. According to Eq (4) to Eq (5) and Eq (7) to Eq (8), the SV index of mobile phone is calculated, and 1378 groups of SV index are obtained. This calculation process is realized by writing SPSS macro program. Limited to space, this paper takes the mobile phone data of the 31st week of 2022 as an example, and takes this week as the base period to explain the SV index compilation process. The calculation results of parameter and SV index are shown in Tables 7 and 8.

**Table 7:** Corresponding parameter of the top 100 mobile phones from the 31st week of 2022 to the 35th week of 2023.

Mobile phone brand	$\varphi_{i}$	Mobile phone brand	$\varphi_{i}$	Mobile phone brand	$\varphi_{i}$	Mobile phone brand	$\varphi_{i}$
HUAWEI	0.0203	MI	0.0241	Apple	0.0052	MI	0.0051
NOKIA	0.0220	OPPO	0.0241	Apple	0.0051	MI	0.0048
NOKIA	0.0184	OPPO	0.0239	Apple	0.0048	MI	0.0047
Apple	0.0150	vivo	0.0238	Apple	0.0044	MI	0.0044
Apple	0.0122	vivo	0.0234	Apple	0.0042	MI	0.0045
Apple	0.0098	vivo	0.0233	Apple	0.0041	MI	0.0043
Apple	0.0076	PHILIPS	0.0226	Apple	0.0042	MI	0.0040
Apple	0.0063	HUAWEI	0.0232	Apple	0.0041	MI	0.0042
Apple	0.0060	HUAWEI	0.0223	Apple	0.0042	MI	0.0041
Apple	0.0063	HUAWEI	0.0228	HONOR	0.0039	MI	0.0037
Apple	0.0063	HUAWEI	0.0232	HONOR	0.0039	MI	0.0034
Apple	0.0070	HUAWEI	0.0216	HONOR	0.0039	MI	0.0033
HONOR	0.0085	HUAWEI	0.0206	HONOR	0.0044	MI	0.0032
HONOR	0.0096	Apple	0.0192	HONOR	0.0043	MI	0.0035
HONOR	0.0112	Apple	0.0169	HONOR	0.0045	MI	0.0035
HONOR	0.0130	Apple	0.0148	HONOR	0.0044	Ace	0.0039
K-Touch	0.0151	Apple	0.0137	HONOR	0.0043	OPPO	0.0039
K-Touch	0.0174	Apple	0.0122	HONOR	0.0042	OPPO	0.0044
MI	0.0184	Apple	0.0107	HONOR	0.0046	OPPO	0.0042
MI	0.0190	Apple	0.0102	HONOR	0.0044	OPPO	0.0044
MI	0.0196	Apple	0.0096	K-Touch	0.0044	vivo	0.0041
MI	0.0214	Apple	0.0079	K-Touch	0.0045	vivo	0.0041
MI	0.0219	Apple	0.0071	K-Touch	0.0045	vivo	0.0042
MI	0.0216	Apple	0.0059	MI	0.0047	vivo	0.0034
MI	0.0230	Apple	0.0052	MI	0.0047	vivo	0.0032

Note: Brand names in the table are based on the 31st week of 2022.

**Table 8:** SV Index based on the 31st week of 2022.

Weeks	SV Index	Weeks	SV Index	Weeks	SV Index
1,2	1.17	1,20	1.31	1,38	1.06
1,3	1.04	1,21	1.15	1,39	1.01
1,4	1.00	1,22	1.19	1,40	0.82
1,5	1.03	1,23	0.99	1,41	1.02
1,6	1.13	1,24	1.08	1,42	1.15
1,7	1.01	1,25	1.03	1,43	1.28
1,8	1.01	1,26	0.99	1,44	1.35
1,9	1.07	1,27	0.97	1,45	1.22
1,10	1.20	1,28	0.98	1,46	1.20
1,11	1.09	1,29	1.11	1,47	1.25
1,12	1.15	1,30	1.14	1,48	1.01
1,13	1.17	1,31	1.02	1,49	1.14
1,14	1.47	1,32	0.91	1,50	1.04
1,15	1.38	1,33	1.08	1,51	1.10
1,16	1.31	1,34	1.14	1,52	1.00
1,17	1.06	1,35	1.00	1,53	0.91
1,18	0.83	1,36	1.21		
1,19	1.03	1,37	1.09		

#### Mobile phone price index calculation based on RYGEKS.

Based on the above SV index, the GEKS price index and the RYGEKS price index are calculated according to Eq (10) to Eq (11). The GEKS ring-to-ring price index based on the window width T = 53 is shown in Table 9. This process is also realized by writing SPSS macro program.

**Table 9:** The window width T = 53 weeks of the GEKS ring price index.

Weeks	GEKS price index	Weeks	GEKS price index	Weeks	GEKS price index
1, 2	0.92	19, 20	1.27	37, 38	0.84
2, 3	0.96	20, 21	0.84	38, 39	1.00
3, 4	0.96	21, 22	0.98	39, 40	0.93
4, 5	0.92	22, 23	1.03	40, 41	1.08
5, 6	0.99	23, 24	1.00	41, 42	1.08
6, 7	1.06	24, 25	0.95	42, 43	1.07
7, 8	1.01	25, 26	0.97	43, 44	1.10
8, 9	1.24	26, 27	1.00	44, 45	0.91
9, 10	1.07	27, 28	1.05	45, 46	1.03
10, 11	0.91	28, 29	1.06	46, 47	0.98
11, 12	1.06	29, 30	0.99	47, 48	0.84
12, 13	1.00	30, 31	0.88	48, 49	1.08
13, 14	1.11	31, 32	1.04	49, 50	0.94
14, 15	1.16	32, 33	1.13	50, 51	1.05
15, 16	1.00	33, 34	0.98	51, 52	0.99
16, 17	0.78	34, 35	0.94	52, 53	0.89
17, 18	0.85	35, 36	1.11		
18, 19	1.21	36, 37	0.98		

It can be seen from Table 9 that most of the values of the GEKS month-on-month price index are between 0.9 and 1.1, that is, the price change between adjacent two weeks is not very large, which is also consistent with common sense, because the ICT products are updated quickly, but measured by week, the price change will not be very large, which also shows the rationality of the price index model constructed in this paper to a certain extent.

According to Table 9 and Eq (10), the fixed base GEKS price index can be calculated, that is $P_{1,53}^{SV-GEKS}=0.97$. Comparing the number with the $P_{1,53}^{SV}=0.91$ in Table 8, it can be seen that the difference between the base index calculated based on the GEKS method with the base period of the first week and the reporting period of the 53rd week and the base index calculated directly using the SV index is 0.06, which is much smaller than the difference between the chain index (5.72) calculated by the 1st and 35th week cycle SV index and the base index calculated directly using the SV index. From this point of view, the fixed-base GEKS price index plays an important role in reducing chain drift.

Taking the window width T = 53 weeks, according to the Eq (10), taking (2,...,54), (3,...,55), (4,...,56) and (5,...,57) as the reference weeks respectively, the ring-to-ring RYGEKS price indexes of the (53,54), (54,55), (55,56) and (56,57) weeks are calculated, which are $P_{53,54;(2,\cdots 54)}^{SV-RYGEKS}=0.99$, $P_{54,55;(3,\cdots ,55)}^{SV-RYGEKS}=0.98$, $P_{55,56;(4,\cdots ,56)}^{SV-RYGEKS}=0.99$ and $P_{56,57;(5,\cdots ,57)}^{SV-RYGEKS}=0.98$ respectively.

Combining the above (53,54), (54,55), (55,56) and (56,57) week-on-week RYGEKS price index with $P_{1,53}^{SV-GEKS}$, the fixed base price index of the 54th, 55th, 56th and 57th weeks relative to the first week is calculated by Eq (11), respectively:


$$P_{1,54}^{SV-RYGEKS}=P_{1,53}^{SV-GEKS}\cdot P_{53,54;(2,\cdots ,54)}^{SV-RYGEKS}=0.96$$

$$P_{1,55}^{SV-RYGEKS}=P_{1,53}^{SV-GEKS}\cdot P_{53,54;(2,\cdots ,54)}^{SV-RYGEKS}\cdot P_{54,55;(3,\cdots ,55)}^{SV-RYGEKS}=0.95$$

$$P_{1,56}^{SV-RYGEKS}=P_{1,53}^{SV-GEKS}\cdot P_{53,54;(2,\cdots ,54)}^{SV-RYGEKS}\cdot P_{54,55;(3,\cdots ,55)}^{SV-RYGEKS}\cdot P_{55,56;(4,\cdots ,56)}^{SV-RYGEKS}=0.96$$

$$P_{1,57}^{SV-RYGEKS}=P_{1,53}^{SV-GEKS}\cdot P_{53,54;(2,\cdots ,54)}^{SV-RYGEKS}\cdot P_{54,55;(3,\cdots ,55)}^{SV-RYGEKS}\cdot P_{55,56;(4,\cdots ,56)}^{SV-RYGEKS}\cdot P_{56,57;(5,\cdots ,57)}^{SV-RYGEKS}=0.95$$

From the above calculation, it can be seen that with the 31st week of 2022 as the base period, the fixed base price index in the 31st week of 2023 is 0.96, indicating that compared with the 31st week of 2022, the overall price level of mobile phones in the 31st week of 2023 will decrease by 4 percentage points. In the 32nd, 33rd, 34th and 35th weeks of 2023, the overall price level of mobile phones decreased by 4%, 5%, 4% and 5%, respectively.

The above calculation and analysis results are based on the construction of the third part of the model. From the perspective of the last week ‘s month-on-month price index and the fixed base price index, it is consistent with the analysis of the theoretical part. The overall price level of the top 100 mobile phones between each week has not changed much. The fixed base index after the 35th week of 2023 shows that the top 100 mobile phones have declined compared with the 31st week of 2022, with a decline of 4%-5%. In addition, according to the time range of the data in this paper, from the perspective of time comparability, the GEKS chain price index from the 31st week to the 35th week of 2022 and the 31st week to the 35th week of 2023 are selected, and the geometric average of the weekly GEKS chain price index in 2022 and 2023 is calculated respectively. The calculation results are 0.9513 and 0.9839 respectively. From this, it can be seen that between August-September 2022 and August-September 2023, from the perspective of weekly average, the mobile phone price index in August 2023 was higher than that in August 2022; from the window width of 53 weeks, there are 22 weeks belonging to 2022 and 31 weeks belonging to 2023 in the sample data. Therefore, based on the GEKS price index for a total of 22 weeks in 2022 and the GEKS price index for a total of 31 weeks in 2023, the geometric average of the weekly GEKS price index in 2022 and 2023 is calculated respectively, which is used as the weekly average price index of mobile phones in 2022 and 2023. The calculation results show that the weekly price index of mobile phones in 2022 and 2023 is 1.0063 and 0.9935 respectively, indicating that the mobile phone price index in 2023 is slightly lower than that in 2022. The ex-factory price index of industrial producers in the computer, communication and other electronic equipment manufacturing industries published by the National Bureau of Statistics in 2022 and 2023 is 1.007 and 0.983, respectively. It can be seen that according to the conclusions calculated from the sample data in this paper, the downward trend of the mobile phone price index in 2023 compared with 2022 is consistent with the trend of the corresponding classification index published by the National Bureau of Statistics. This also shows to a certain extent that the basic classification price index model constructed in this paper has certain rationality.

In summary, based on the Hedonic-SV-RYGEKS model constructed in the previous section of this section, the Hedonic-Robust/ Huber-SV-RYGEKS regression model is specifically selected. Taking the preparation of mobile phone price index as an example, the empirical research on the constructed model is carried out. The research results show that the mobile phone price index based on this model not only considers the elasticity of substitution of products, but also reflects the change of mobile phone quality, and plays a positive role in reducing the chain drift of price index, which can better reflect the actual change of mobile phone price.

## An empirical study on the compilation of notebook computer price index based on Hedonic-SV-RYGEKS index

In order to further test the rationality of the model construction in this paper, this section selects the basic classification of notebook computers in ICT products as the research object and compiles its corresponding price index.

### Data sources and data processing

Similar to the difficulty of obtaining mobile phone data in the previous section, it is also difficult to obtain specific data related to the price of notebook computers. In view of the cost of data acquisition, the data in this section is realized through the following process. First of all, this paper collects the basic information of the title, sales volume, sales volume and average price of the top 20 products in the domestic notebook computers from August 2023 to December 2024 in the “Mirror Insight”; secondly, the SAS software is used to eliminate the repeated commodity information in the month, and the R software is used to extract the top 60 commodities in the descending order of sales volume in the month, so as to ensure the consistency of the sample number between months. Thirdly, according to the keywords listed in the title of the product, python software is used to obtain the specific feature information corresponding to the same or similar products of these products on the Jingdong website ‘crawler’; fourth, find and supplement some missing information in the above crawler data, and finally form the basic data for calculating the price index.

Since the data used in this section is monthly data, in terms of window width selection, the window width is selected to be 13 months, that is, the first 13 months of the 17 months are used as window widths, and the remaining 4 months are used for rolling calculation of the fixed base price index. The compilation process of the Notebook computer price index is similar to that of the previous mobile phone price index. As mentioned above, the hedonic model has three forms. In the previous section, the linear model is selected to compile the mobile phone price index. In this section, the semi-logarithmic hedonic model is selected to further test the applicability of the Hedonic-SV-RYGEKS price index model constructed in this paper.

The semi-logarithmic Hedonic model is used to adjust the price of mismatched notebook computers for any two months to obtain a comparable notebook computer price sequence for any two months. The specific process of this data processing can refer to the preparation process of the mobile phone price index in the previous section. The difference is that the selection of the Hedonic model and its parameter estimation method. Here, a semi-logarithmic hedonic regression model is established for the 17-month notebook computer price and the notebook computer characteristic variable, and the characteristic regression equation of the monthly notebook computer price is obtained. In this process, the weighted least squares method is used to estimate the model parameters. After the above process, the sample size of the notebook computer each month is still 60, and the notebook computers of any two months are comparable after quality adjustment.

### Notebook computer price index compilation based on Hedonic-SV-RYGEKS method

#### Mismatch item processing based on semi-logarithmic Hedonic model.

Considering the robustness of the model, the data set is divided into two parts: training set and test set according to 4: 1. According to Eq (1), the average price of notebook computers in 13 months and its related characteristic variables (characteristic variables include brand (Mechrevo (jx)、Lenovo (lx)、ASUS (hs)、dell、others (qt)), CPU model (CPU), memory capacity (me), graphics card type (card), hard disk capacity (hard), screen characteristics (screen) and screen size (size)) are regressed and modelled. The Breusch-Pagan test and White test are used to test the heteroscedasticity of the model. For the model that does not pass the test, the weighted least squares method is used for regression. Here the model does not consider the constant term. The estimation of 17 groups of equations is realized by writing Python program. The goodness of fit, equation significance test, autocorrelation test and heteroscedasticity test of semi-logarithmic Hedonic regression equation are shown in Table 11. The corresponding regression coefficients of the 17 groups of equations established by passed the test at the significance level α = 0.1, and the results are shown in Table 10. From Table 11, it can be seen that the goodness of fit of the regression model in this 17 month is above 0.7. Therefore, the overall model fitting effect is better, the equation passes the significance test, there is no heteroscedasticity and auto-correlation, and the model training set and the test set MSE is not much different.

**Table 10:** Parameter estimation of semi-logarithmic Hedonic model for each month from August 2023 to December 2024.

eq	jx	lx	hs	dell	qt	CPU	me	card	hard	screen	size
1	8.7790	8.9730	9.1600		8.6850			-0.5610		0.3730	
2	8.9625	9.2608	9.0776	9.0999	8.9423	0.0794	-0.0126	-0.4168	0.0006	-0.0287	-0.0352
3	8.5520	8.7660	8.7500	8.4950	8.5840			-0.4070	0.0030		
4	1.6620	1.6680	1.9400	2.0280	1.6780	-0.0560					0.4560
5	1.2470	1.2610	1.6100	1.2920	1.2100		0.2440				0.4540
6	3.8440	3.9420	4.4110	3.9190	3.9440		0.0068	-0.1470	0.0030		0.2823
7	10.9100	11.0520	10.9910	10.9000	10.7500	-0.1274		-0.4240	0.0003	-0.0950	-0.1420
8	9.2930	9.5280	9.3720	9.5280	9.4580			-0.3550	0.0002	0.0582	-0.0493
9	9.9957	10.1031	9.9599	10.0687	10.0514		-0.0047	-0.4199	0.0002	0.1215	-0.0847
10	8.3792	8.5750	8.5985	8.4318	8.3709		0.0143	-0.4496	0.0002		
11	10.0542	10.2057	10.2249	10.1517	9.9985		0.0050	-0.5633	0.0004	0.0495	-0.1015
12	10.1207	10.5470	10.4387	10.4560	10.2659	-0.0776		-0.3557	0.0007	-0.1242	-0.1308
13	8.5078	8.6548	8.7914	8.6372	8.4831		0.0117	-0.5539	0.0001	0.1608	
14	7.5147	7.6773	7.7729	7.4358	7.4613	0.0811	0.0136	-0.4468	0.0003	0.2802	0.0493
15	8.3728	8.5579	8.5708	8.7396	8.3361		0.0123	-0.5255	0.0003	-0.1696	
16	11.4935	11.5268	11.6948	11.6124	11.4821	0.0683		-0.4475	0.0002	-0.5150	-0.1794
17	9.8841	10.3784	10.2582	10.3188	9.7920	-0.0940	-0.0079	-0.5164	0.0005	-0.3303	-0.8870

**Table 11:** Monthly semi-logarithmic Hedonic model test from August 2023 to December 2024.

id	Adj. R-squared	F-statistic	D-W-statistic	bp_lm_pval	bp_F_pval	JB_pval	Training MSE	Testing MSE
1	1.000	2.51E + 05	2.205	0.053	0.079	0.888	0.022	0.020
2	0.998	2387.000	2.102	0.072	0.055	0.073	0.023	0.025
3	0.981	398.800	2.123	0.040	0.032	0.062	0.028	0.027
4	0.929	104.100	1.887	0.112	0.110	0.074	0.085	0.136
5	0.983	453.000	1.962	0.556	0.592	0.078	0.113	0.126
6	0.778	21.630	2.326	0.167	0.167	2.627	0.068	0.065
7	0.879	38.200	1.692	0.470	0.512	0.000	0.045	0.139
8	0.997	2255.000	2.049	0.800	0.837	0.084	0.034	0.039
9	0.986	372.900	1.865	0.159	0.156	0.192	0.046	0.106
10	0.995	1193.000	2.033	0.663	0.704	0.166	0.031	0.049
11	1.000	22910.000	2.023	0.060	0.046	0.104	0.025	0.110
12	0.958	120.900	2.314	0.863	0.896	0.052	0.046	0.030
13	0.982	313.000	2.825	0.072	0.060	0.135	0.033	0.054
14	0.996	934.600	2.362	0.168	0.164	0.111	0.073	0.042
15	0.993	896.900	1.944	0.064	0.053	0.127	0.039	0.033
16	0.995	1109.000	1.621	0.268	0.283	0.143	0.020	0.144
17	0.950	89.690	1.700	0.673	0.726	0.126	0.056	0.108

Based on the trained model, the characteristic variables of the base period mismatched notebook computer are brought into the report period model, and the price of the base period mismatched notebook computer in the report period is calculated, which lays a foundation for the later notebook computer price compilation.

#### Robustness test of semi-logarithmic Hedonic model.

**Fig 1:**
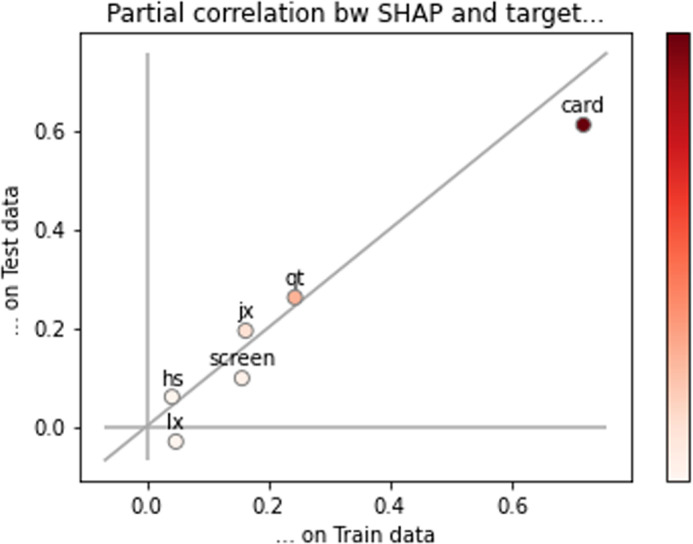
Comparison of ParShap values between training set and test set (August 2023).

**Fig 2:**
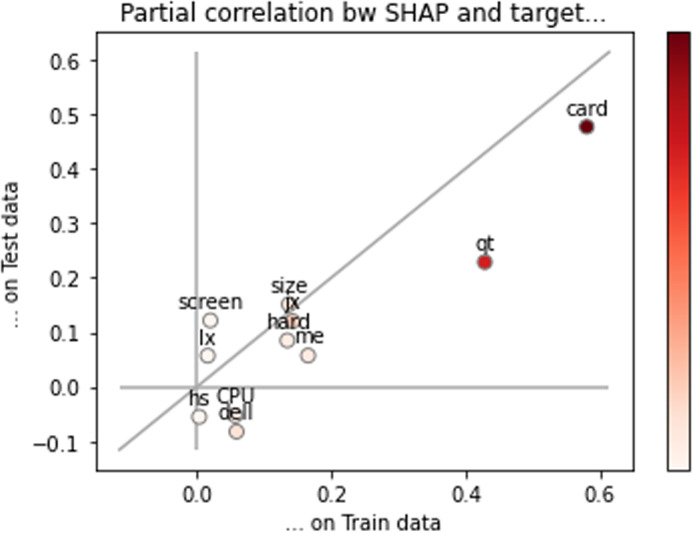
ParShap value comparison between training set and test set (December 2024).

The mean square error of the training set and the test set in Table 11 shows that the semi-logarithmic Hedonic model of the notebook computer price in this section is relatively stable. In order to further verify the robustness of the model, the XGboost linear model is used for comparison. The specific practice is as follows: based on the variables used in the trained semi-logarithmic Hedonic model, the data set is still divided into training set and test set according to 4: 1, and the XGboost linear regression model is realized by python. Finally, the model prediction effect is seen by calculating the ParShap values of the training set and the test set. Taking the data sets of August 2023 and December 2024 as examples, the relationship between the ParShap values of the training set and the test set is shown in Figs 1 and 2. It can be seen from Figs 1 and 2 that most of the variables in the XGboost model are near the diagonal. From this point of view, it is reasonable to choose the linear model as the prediction model, which further shows that the selection of the linear model in this section is correct.

#### Notebook computer price index calculation based on RYGEKS.

It can be seen from Table 12 that most of the monthly price index of GEKS is between 0.9 and 1.1. According to Table 12, the quarterly price index of notebook computer is further calculated. Here, the quarterly price index is calculated in the form of geometric average. After calculation, the notebook computer price index in the fourth quarter of 2023, the first quarter of 2024, the second quarter of 2024 and the third quarter of 2024 is 1.003, 0.9732, 1.0091 and 1.0340, respectively. According to the China Notebook Computer Online Retail Market Monthly Tracker released by RUNTO, since the second quarter of 2023, the average online price of notebook computers in China has shown a trend of “first suppression and then rise”. The average price hit the bottom in the first quarter of 2024, and then the average price began to rise. By the third quarter of 2024, the average price reached CNY 6472. It can be seen that the notebook computer price index calculated by the price index model constructed in this paper is consistent with the actual notebook computer average price change trend, which shows the rationality of the price index model constructed in this paper.

**Table 12:** Window width T = 13 Month Notebook computer GEKS chain price index.

Month	GEKS price index	Month	GEKS price inde	Month	GEKS price index
1, 2	1.01	5, 6	0.96	9, 10	1.07
2, 3	0.99	6, 7	0.99	10, 11	0.97
3, 4	1.03	7, 8	0.97	11, 12	1.13
4, 5	0.99	8, 9	0.99	12, 13	0.90

According to Table 12 and Eq (10), the fixed base GEKS price index can be calculated, namely $P_{1,13}^{SV-GEKS}=0.9856$. Comparing this number with that $P_{1,13}^{SV}=1.0168$ in Table 8, the difference between the two is 0.0312. At the same time, it can also be seen that the base index calculated based on the GEKS method with the first month as the base period and the 13 th month as the reporting period shows that the average price of notebook computers in August 2024 is 1.4% lower than that in August 2023, while the base index calculated directly using the SV index shows that the average price of notebook computers in the corresponding month increased by 1.2%. On the other hand, the chain index of the first month and the 13th month is calculated according to the chain SV index from the first month to the 13th month, 0.8022. Obviously, this value is quite different from the fixed base index (1.0168) calculated directly by SV index. From this point of view, the fixed-base GEKS price index plays an important role in reducing chain drift. In addition, according to the data of the producer price index of the computer, communication and other electronic equipment manufacturing industry in the industrial producer price index published by the National Bureau of Statistics of China, the producer price index of the computer, communication and other electronic equipment manufacturing industry in August 2024 showed that the price of the index category fell by 2.4% year-on-year, while the above-mentioned fixed-base GEKS price index fell by 1.4%, and the price change trend of the category was consistent. This further illustrates the advantages of the model constructed in this section in calculating the fixed base price index.

Taking the window width T = 13 months, according to Eq (10), taking (2,...,14), (3,...,15), (4,..., 16) and (5,...,17) as reference months, the month-on-month RYGEKS price indexes of (13,14), (14, 15), (15,16) and (16,17) are calculated as follows: $P_{13,14;(2,\cdots 14)}^{SV-RYGEKS}=1.0160$, $P_{14,15;(3,\cdots ,15)}^{SV-RYGEKS}=0.9837$, $P_{15,16;(4,\cdots ,16)}^{SV-RYGEKS}=0.9751$ and $P_{16,17;(5,\cdots ,17)}^{SV-RYGEKS}=0.9912$.

Combining the above month-on-month RYGEKS price index for the months (13,14), (14,15), (15,16) and (16,17) with $P_{1,13}^{SV-GEKS}$, the fixed base price indexes for the months 14,15,16 and 17 relative to January are calculated by Eq (11), which are:


$$P_{1,14}^{SV-RYGEKS}=P_{1,13}^{SV-GEKS}\cdot P_{13,14;(2,\cdots ,14)}^{SV-RYGEKS}=1.0014$$

$$P_{1,15}^{SV-RYGEKS}=P_{1,13}^{SV-GEKS}\cdot P_{13,14;(2,\cdots ,14)}^{SV-RYGEKS}\cdot P_{14,15;(3,\cdots ,15)}^{SV-RYGEKS}=0.9887$$

$$P_{1,16}^{SV-RYGEKS}=P_{1,13}^{SV-GEKS}\cdot P_{13,14;(2,\cdots ,14)}^{SV-RYGEKS}\cdot P_{14,15;(3,\cdots ,15)}^{SV-RYGEKS}\cdot P_{15,16;(4,\cdots ,66)}^{SV-RYGEKS}=0.9640$$

$$P_{1,17}^{SV-RYGEKS}=P_{1,13}^{SV-GEKS}\cdot P_{13,14;(2,\cdots ,14)}^{SV-RYGEKS}\cdot P_{14,15;(3,\cdots ,15)}^{SV-RYGEKS}\cdot P_{15,16;(4,\cdots ,66)}^{SV-RYGEKS}\cdot P_{16,17;(5,\cdots ,17)}^{SV-RYGEKS}=0.9556$$

From the above calculation, it can be seen that with August 2023 as the base period, the fixed base price index in September 2024 was 1.0014, indicating that the price level of notebook computers in September 2024 increased by about 0.1 percentage points compared with August 2023; In October, November and December 2024, the overall price level of notebook computers decreased by 1.13%, 3.6% and 4.44%, respectively.

The above calculation and analysis results are carried out on the basis of the model construction in the third part. From the last month ‘s chain price index and fixed base price index values, it is consistent with the theoretical analysis. The overall price level of the top 60 notebook computers in each month has not changed much. The fixed base index after August 2024 shows that the top 60 notebook computers are generally lower than August 2023, with a decrease of 1% -4%. In addition, according to the time range of the data in this paper, the monthly GEKS month-on-month price index in the fourth quarter of 2023 and the fourth quarter of 2024 is selected from the perspective of time comparability, and the geometric mean of the monthly GEKS month-on-month price index is calculated respectively. The calculation results are 1.003 and 0.9995 respectively. It can be seen that in the fourth quarter of 2023 and the fourth quarter of 2024, from the perspective of monthly average, the notebook computer price index in 2024 is lower than that in 2023. In the same way, using the ‘Computer, Communication and Other Electronic Equipment Manufacturing Industry Producer Price Index’ in the monthly industrial producer price index of 2023 and 2024 published by the National Bureau of Statistics of China, the monthly month-on-month price index of the fourth quarter of 2023 and the fourth quarter of 2024 was calculated to be 0.9993 and 0.9990, respectively. It can be seen that according to the conclusion calculated from the sample data in this paper, the notebook computer price index in the fourth quarter of 2024 has a downward trend compared with the fourth quarter of 2023, which is consistent with the trend of the corresponding classification index published by the National Bureau of Statistics. This also shows to a certain extent that the basic classification price index model constructed in this paper has certain rationality.

In summary, this section is based on the Hedonic-SV-RYGEKS model constructed in the second section. In order to further verify the applicability and robustness of the model, this section takes another ICT product-notebook computer price index preparation as an example, which is different from the linear Hedonic model of mobile phone price index preparation. This section selects the semi-logarithmic Hedonic model and uses the weighted least squares method to process the heteroscedasticity. The other process is similar to the preparation of mobile phone price index. This price index preparation model is called ‘Hedonic-WLS-SV-RYGEKS’. The research results show that the notebook computer price index based on this model, like the previous mobile phone price index, not only considers the elasticity of product substitution, but also reflects the change of mobile phone quality, and plays a positive role in reducing the chain drift of price index, which can better reflect the actual change of notebook computer price. At the same time, compared with the process of mobile phone price quality adjustment, it is further explained that the flexibility of Hedonic model establishment, linear model and semi-logarithmic model need to be considered in combination with data. From the perspective of the price index of mobile phones and notebook computers in this paper, the three forms of the Hedonic model mentioned above in the Hedonic-SV-RYGEKS model can be selected according to the model and data.

In short, this paper takes the price index compilation of mobile phones and notebook computers in ICT products as an example, and empirically tests the constructed Hedonic-SV-RYGEKS model from different levels. Whether it is weekly data or monthly data, the price index compiled based on the Hedonic-SV-RYGEKS model can better reflect the price changes of ICT products.

## Research conclusions and prospects

### Conclusions

In the era of digital economy, GEKS price index calculation method has become a relatively new field in price index theory, which has been used in some countries around the world. In this paper, from the perspective of ICT product quality adjustment and product substitutability, the SV index considering the elasticity of substitution of products is selected for the construction of the basic price index, and on this basis, the Hedonic-SV-RYGEKS price index theoretical model is constructed, and further theoretical and applied research is carried out. The following important conclusions are obtained:

Firstly, this paper constructs the Hedonic-SV-RYGEKS price index model with reference to the suggestion of ‘Consumer Price Index Manual: Concepts and Methods (2020)’. The model combines the characteristics of the quality adjustment model to avoid the sample incomparability caused by the low matching degree of inter-temporal samples. The elasticity of substitution between products is considered; at the same time, it effectively suppresses the chain drift problem of the chain price index caused by the rapid product update. In theory, this model is a more reasonable price index compilation model, which can provide some reference ideas for solving the sample incomparability caused by the high loss rate of products in the compilation of CPI basic classification index in the digital economy era, and improving the accuracy of price index compilation.

Secondly, based on the constructed Hedonic-SV-RYGEKS model, the mobile phone and notebook computer price indexes are compiled. Specifically, taking the price index compilation of mobile phones and notebook computers as an example, the linear Hedonic-Robust/ Huber model and the semi-logarithmic Hedonic-WLS model are established respectively by obtaining the online price data of the corresponding frequency through ‘crawler’. The difference between the two models lies in the different selection forms of the Hedonic model and the different treatment methods for the data heteroscedasticity phenomenon. Based on the linear Hedonic-Robust/ Huber model and the semi-logarithmic Hedonic-WLS model, the Hedonic-SV-RYGEKS price index of mobile phones and notebook computers is compiled. The research results show that the price index compiled based on the online data obtained by the crawler in this paper is similar to the trend of the industry classification price index of the National Bureau of Statistics of China, which further verifies the correctness of the model construction from an empirical perspective. This may also indicate that the model in this paper may provide some practical references for national institutions in compiling corresponding product price indexes. At the same time, from the perspective of the data used in this paper, only a small amount of sample information that can be obtained for free ‘crawler’ is used. For example, the sample size of mobile phones is 100, and the sample size of notebook computers is 60. Using these free data combined with the model in this paper, we can get a trend similar to the actual change of the product, which further shows that the compilation of the price index of mobile phones and computers in this paper can also provide reference for individuals or enterprises to make decisions on ICT products.

Thirdly, the model constructed in this paper has the characteristics of strong operability and easy interpretation. This can be confirmed by the establishment of the Hedonic model. This model generally belongs to a linear regression model, which is simple and easy to operate, and is easier to explain than the machine learning model. In short, the Hedonic-SV-RYGEKS price index model constructed in this paper is more suitable for fast-updating products. Using this model, more detailed product feature information needs to be obtained, which can be extended to other categories. The preparation of ICT product price index with these characteristics.

### Prospects

In recent years, with the rapid development of science and technology, ICT products emerge in an endless stream, and the speed of upgrading is very fast. People use electronic products more and more widely, which provides a great opportunity to improve and innovate the method of dealing with mismatched items in the compilation of price index. Based on the data of related characteristic variables of mobile phones and notebook computers obtained by whale staff platform and magic mirror insight combined with ‘crawler’, this paper tries to sort out a solution to the problems of rapid upgrading of ICT products and elasticity of substitution between products, hoping to play a reference role in the follow-up research.

The research method of this paper can be further extended to the compilation of ICT product price index with faster upgrading other than mobile phones and notebook computers. Due to the limitation of the data used in this paper, the price index calculation is not done for a longer time. Although the model in this paper can better reflect the changes in the prices of mobile phones and notebook computers, there is also room for improvement. For example, in the process of establishing the Hedonic model, for the processing of data heteroscedasticity, for mobile phones, this paper chooses Robust or Huber regression based on OLS regression. The follow-up study can consider comparing with the direct Robust or Huber regression model, or using some machine learning models for comparative study. In addition, in the preparation of the RYGEKS index, in terms of window width selection, the window width of 53 weeks is selected for weekly data, and the window width of 13 months is selected for monthly data. There is no more window width length setting, and the window width selection will be discussed in the follow-up study. When conditions permit, offline and online data can be combined to discuss, so as to enrich the data sources of price index research in this paper.

## Supporting information

S1 File Partial raw data. (ZIP)
